# Precision distance control in ultrasound-guided coaxial needle biopsy for small liver cancer: improving safety and diagnostic accuracy yield

**DOI:** 10.3389/fonc.2026.1728052

**Published:** 2026-03-18

**Authors:** Yaqi Zhang, Qian Huang, Lilin Zhao, Ting Zhang

**Affiliations:** Department of Ultrasound, The Affiliated Cancer Hospital of Nanjing Medical University, Jiangsu Cancer Hospital, Jiangsu Institute of Cancer Research, Nanjing, Jiangsu, China

**Keywords:** coaxial needle, precision distance control, procedure - related complications, small component, small liver cancer

## Abstract

**Objective:**

The diagnostic yield of percutaneous biopsy for small liver cancer is affected by lesion size and location. This study utilizes small components with a coaxial cannula to enables precise control over specimen length, aiming to evaluate the safety and clinical applicability of ultrasound-guided coaxial needle biopsy.

**Methods:**

In the coaxial group, the biopsy gun’s position relative to the coaxial cannula was adjusted, and small components were used to control specimen length. Thrombin (1–2 KU) was injected along the needle tract post-procedure. In the non-coaxial group, multiple needle insertions targeted the lesion edge. Complications and pathology results were recorded to assess clinical efficacy.

**Results:**

A retrospective analysis of 744 patients from Jiangsu Provincial Cancer Hospital showed a higher pathological detection rate in the coaxial group (95.6%, 198/207) than in the non-coaxial group (89%, 478/537). Intraoperative bleeding was more easily detected in the coaxial group, allowing timely intervention. Pain was reported in 15% (31/207) versus 30% (161/537), respectively. No needle tract implantation occurred in either group.

**Conclusion:**

Ultrasound-guided coaxial needle biopsy for small liver cancer (≤2 cm) significantly improves diagnostic accuracy (P = 0.005), reduces procedure complexity and complications, and enhances patient outcomes, demonstrating excellent safety and clinical value.

## Introduction

The latest data released by the National Cancer Center of China shows that liver cancer ranks fourth among new cases of malignant tumors and second in mortality rate, posing a serious threat to public health and life survival (1). Early diagnosis and treatment are crucial for improving patient prognosis (2). While liver neoplasms larger than 2 cm in diameter can be directly diagnosed through typical imaging features, the diagnostic sensitivity for small hepatic lesions (≤ 2 cm in diameter) is only 33% by imaging alone (3), necessitating confirmation via biopsy (4). This diagnostic imperative is particularly critical for subcentimeter nodules, where contemporary evidence still indicates a non-negligible risk of hepatocellular carcinoma, reinforcing the need for definitive tissue diagnosis when imaging is inconclusive (5). Percutaneous biopsy of small liver lesions, however, is fraught with challenges, including challenging lesion location, respiratory motion, and the inherent difficulty in obtaining sufficient tissue from smaller tumors.

Ultrasound-guided percutaneous biopsy serves as the primary diagnostic modality for these lesions. This diagnostic imperative is supported by the established safety profile of the procedure; a large-scale meta-analysis has confirmed that ultrasound-guided percutaneous liver biopsy carries a very low risk of major complications, affirming its viability as a routine diagnostic approach (6). In practice, two principal technical approaches are utilized: the conventional (non-coaxial) freehand method and the coaxial technique. The conventional method typically requires multiple penetrations of the liver capsule to obtain adequate tissue, which may increase procedural time and the cumulative risk of complications. A contemporary meta-analysis underscores that percutaneous liver biopsy carries a measurable risk, with major and minor complication rates reported at approximately 2.44% and 9.53%, respectively, highlighting the ongoing need for technical refinement to maximize safety (7). In contrast, the coaxial technique employs an indwelling cannula to establish a stable tract, enabling the acquisition of multiple tissue samples through a single capsular puncture. This fundamental difference underpins the theoretical advantages of coaxial biopsy. A recent meta-analysis investigating needle-tract seeding reveals that, while the overall incidence remains low (approximately 1%), the implementation of a coaxial technique is correlated with further risk mitigation compared to the conventional non-coaxial approach (8, 9). The evolution of liver biopsy is increasingly focused on greater precision and standardization, integrating technological refinements to improve diagnostic outcomes (10).

However, both techniques share a common limitation: the mechanical advancement of the biopsy needle lacks standardized control. This results in millimeter-level uncertainty in the final sampling length at the precise moment of tissue cutting. For small tumors adjacent to critical blood vessels or the hepatic capsule, this uncertainty can directly lead to insufficient sampling, compromising the diagnostic yield, while simultaneously increasing procedural risks.

To ruduce this inherent uncertainty, we devised a precision distance control (PDC) strategy within the coaxial biopsy workflow. This approach is designed to standardize the needle’s penetration depth, thereby enhancing the precision and reproducibility of tissue acquisition.

Consequently, this study was designed to evaluate the integrated clinical performance of a coaxial system incorporating this PDC protocol. We aimed to determine whether this enhanced technique demonstrates superior diagnostic accuracy and a better safety profile compared to the conventional non-coaxial approach in patients with small liver cancers (≤2 cm).

## Materials and methods

### Patient selection

A retrospective analysis was conducted on patients undergoing ultrasound-guided percutaneous biopsy of small liver cancer (diameter ≤2 cm) at Jiangsu Provincial Cancer Hospital, from January 2021 to March 2024. All patients received comprehensive imaging evaluations, including ultrasound, CT, and MRI, prior to the biopsy. The primary objective of the biopsy was to ascertain the nature of the lesion and the molecular classification of liver cancer. This information was intended to guide the selection of subsequent surgical approaches, chemotherapy regimens, and prognosis assessment. Informed consent was obtained from all patients prior to the ultrasound-guided biopsy procedure.

This study included 744 patients who met the inclusion criteria, with 207 undergoing biopsy with coaxial needle assistance. All procedures were performed under ultrasound guidance by senior attending physicians or associate chief physicians in the ultrasound department, each with over 10 years of clinical experience. Patients were randomly assigned to each group. There were no differences in the diameters and locations of the lesions between the two groups (the coaxial needle group and the non-coaxial needle group). The cohort consisted of 431 males and 313 females, aged 17 to 94 years.

Inclusion criteria:

Patients undergoing ultrasound-guided percutaneous biopsy of small liver cancer (diameter ≤2 cm).

Exclusion criteria:

- Patients with severe cardiac, renal, hepatic, or other organ dysfunctions that contraindicate biopsy;
- Patients who had taken oral anticoagulants within one week preceding the biopsy;
- Patients with coagulation or immune system disorders;
- Patients whose tissue specimens were sent to external institutions for analysis.

### Equipment and methods

#### Equipment

The Super Sonic Aixplorer, Esaote MyLab9XP and Samsung R10 color Doppler ultrasound machines with a probe frequency of 2–5 MHz. Additionally, an 18G automatic biopsy device from Beijing Demeter and the Bard Medical Technology C1816A coaxial trocar were employed.

#### Preoperative preparation

A comprehensive set of standard preoperative assessments was undertaken for all patients, including complete blood count, coagulation profile, biochemical tests, and electrocardiogram, in order to rule out any contraindications. Patients were instructed to fast for 6–8 hours before the biopsy and were informed about the puncture process to alleviate anxiety.

#### Puncture procedure

Prior to the biopsy, a routine ultrasound was performed alongside other imaging modalities to assess the size, number, shape, echogenicity, and precise location of the lesion, as well as its proximity to major blood vessels and critical organs. This allowed for a preliminary diagnosis. Concurrently, the ultrasound and Doppler characteristics of the lesion were evaluated to select the optimal puncture route.

**Figure 1:**
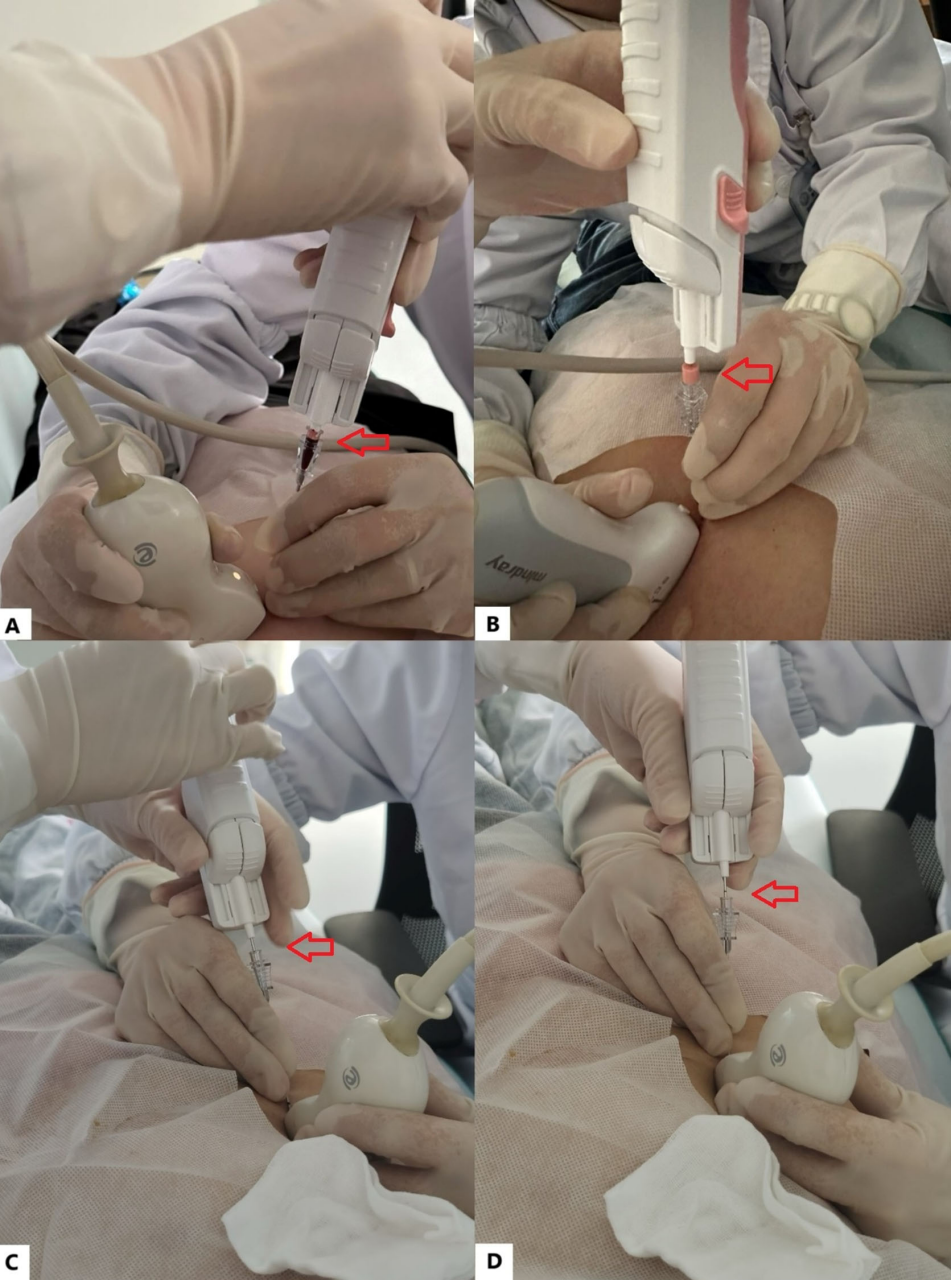
Schematic diagram of the operation of a biopsy needle combined with a coaxial trocar for precise, distance-controlled puncture of small liver cancer: As indicated by the arrows **(A–D)**, the length of the biopsy gun inserted into the coaxial cannula is precisely controlled, allowing for biopsy specimen lengths of 1.4 cm, 1 cm, and 0.5 cm. In this setup, B applies a small section with a length and diameter of 6 mm.

In the combined coaxial needle puncture group, the biopsy procedure was performed as follows: depending on the lesion location, patients were positioned supine or in the left lateral position. After routine disinfection and sterile draping, the optimal puncture entry site was chosen under ultrasound guidance, and the puncture angle and trajectory were determined. After local anesthesia with 5 ml 2% lidocaine, a coaxial needle was inserted in plane along the anesthetic site under ultrasound guidance. The needle tip was advanced to the edge of the lesion, and the inner stylet was removed to create a biopsy channel. The tissue specimen length was precisely controlled by adjusting the insertion depth of the biopsy needle into the coaxial trocar. This adjustment was achieved using small accessory spacers (each 0.6 cm in length), which were inserted into the biopsy gun before needle placement. By selecting the appropriate number of spacers according to the desired specimen length, the operator could ensure accurate control and reduce errors from hand tremor or needle forward movement. Multiple specimens were obtained from different angles and locations (**Figure 1**). After specimen collection, 1–2 KU of thrombin was injected into the biopsy tract to seal the puncture channel and prevent intratumoral or hepato-peritoneal hemorrhage. The cannula core was then reinserted, and the coaxial needle was withdrawn.

In the conventional puncture group, patients were positioned supine or in the left lateral position based on lesion location. After routine disinfection and sterile draping, the optimal puncture site, angle, and trajectory were identified under ultrasound guidance. Following local anesthesia with 5 mL of 2% lidocaine, the handheld biopsy needle was repeatedly advanced to the lesion edge for tissue sampling.

#### Postoperative treatment

Tissue samples from both groups were promptly fixed in 10% formalin, with patient information and specimen site clearly labeled for pathological and histological examination. Compression was then applied to the puncture site to achieve hemostasis; the wound was disinfected and bandaged. Patients were monitored in the hospital for a two-hour postoperative period. In cases of significant postoperative bleeding, hemostatic treatment was administered as required.

### Data collection

Combined Coaxial Trocar Puncture Group: Intraoperative bleeding was diagnosed if rapid blood flow was observed through the coaxial trocar needle. In the conventional puncture group, intraoperative hemorrhage was identified by ultrasound detection of bleeding along the puncture path or a significant increase in peritoneal fluid, as blood could not escape through an open channel. Postoperative hemorrhage criteria were the same for both groups and included persistent bleeding on ultrasound, continuous abdominal fluid accumulation, sudden hypotension, or significant hemoglobin reduction.

The occurrence of bleeding, hemostatic measures, and outcomes were recorded for all groups. Additionally, intraoperative and postoperative pain events, along with their relief measures, were documented. Routine postoperative imaging was conducted at intervals of 3 to 6 months, and the formation of new lesions along the needle path was noted.

### Postoperative assessment

For all patients, the presence and intensity of postoperative pain were prospectively recorded by the attending physician or nurse as part of our standardized clinical audit for this procedure. Pain was assessed at the end of the 2-hour post-procedural observation period using the Numerical Rating Scale (NRS), where patients verbally reported a number from 0 (no pain) to 10 (worst imaginable pain). Based on the NRS scores, pain was categorized into three grades for analysis: no pain (NRS = 0), mild pain (NRS 1-3), and moderate-to-severe pain (NRS ≥ 4). For the analysis of overall pain incidence, a score of NRS ≥ 1 was considered indicative of any postoperative pain.

### Pathology results

Pathology results were considered non-diagnostic if a definitive pathological diagnosis could not be reached. The causes of diagnostic failure were investigated, including lesion size and the patient’s underlying liver disease.

### Statistical analysis

Data were analyzed using SPSS version 27.0. Continuous variables with a normal distribution are presented as mean ± standard deviation (SD) and were compared between the two groups using the independent Student’s t-test. Non-normally distributed continuous variables or ordinal data are presented as median with interquartile range (IQR) and were compared using the Mann-Whitney U test. Categorical variables are presented as frequencies and percentages (%) and were compared using the Chi-square (X²) test or Fisher’s exact test, as appropriate. A two-sided P value of < 0.05 was considered statistically significant. The P value represents the probability of observing the given difference, or a more extreme one, assuming that the null hypothesis is true.

## Results

### Comparison of baseline characteristics

Baseline characteristics were well-balanced between the two groups, as detailed in **Table 1**. No statistically significant differences were observed in age, gender, BMI, lesion diameter, number, or high-risk location profiles (all P > 0.05).

**Table 1:** Baseline characteristics of patients with small liver cancers (≤2 cm) undergoing biopsy.

Characteristic	Combined coaxial trocar puncture group(n=207)	Conventional puncture group (n=537)	P Value
Age (years)	62(1 5)	61(17)	0.936
BMI (kg/m²)	25.16 (4.77)	24.78 (4.35)	0.609
Lesion Size (cm)	1.31 (0.59)	1.43 (0.60)	0.135
Gender, n (%)			0.679
Male	117(56.5%)	314(58.5%)	
Female	90(43.5%)	223(41.5%)	
Lesion Number, n (%)			1.000
Single	85(41.1%)	220(41.0%)	
Multiple (≥2)	122(58.9%)	317(59%)	
High-risk Location, n (%)			
Near Vessel	95(45.9%)	240(44.7%)	0.805
Near Capsule	58(28.0%)	169(31.5%)	0.376

Data are presented as median (interquartile range) for continuous variables, compared using the Mann-Whitney U test; and as number (percentage) for categorical variables, compared using Fisher’s exact test.

#### Puncture outcomes

**Figure 2:**
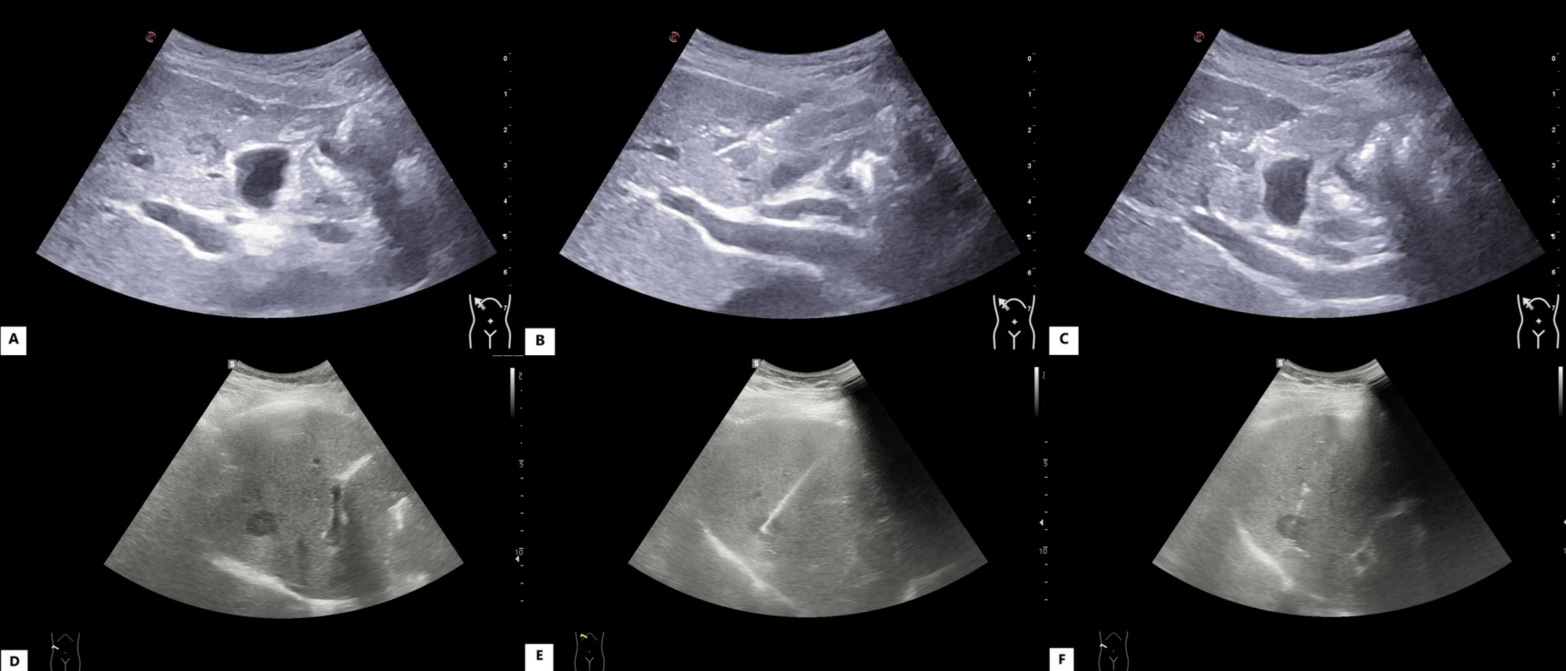
Preoperative, intraoperative, and postoperative ultrasound images of small liver cancer puncture using combined coaxial trocars in patients with different lesion sizes. **(A–C)** Images from a patient with a lesion diameter ≤1 cm: **(A)** preoperative, **(B)** intraoperative, **(C)** postoperative. **(D–F)** Images from a patient with a lesion diameter of 1 < d ≤ 2 cm: **(D)** preoperative, **(E)** intraoperative, **(F)** postoperative.

**Figure 3:**
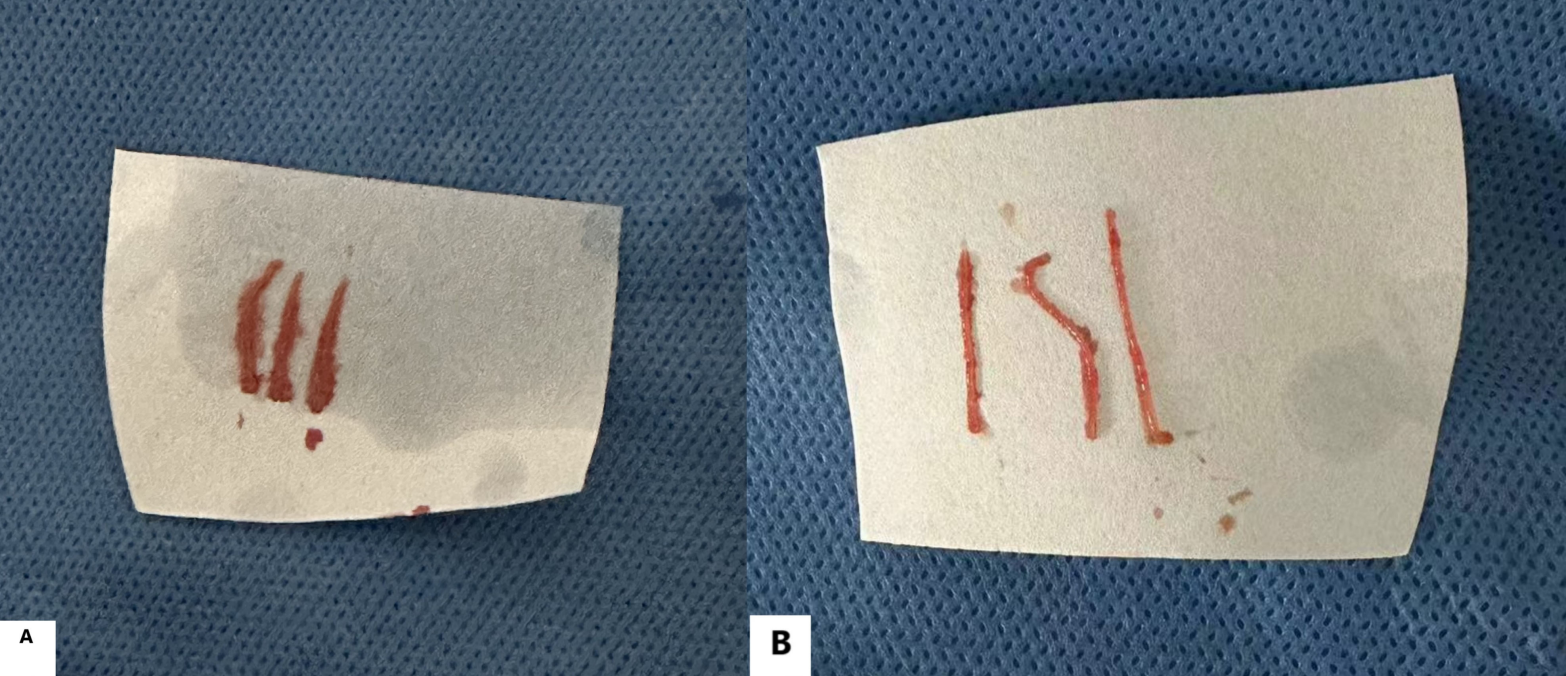
Pathological tissue samples obtained through biopsy using combined coaxial trocars in patients with different lesion sizes: **(A, B)** show complete, long strips of lesion tissue removed by puncture.

Combined coaxial trocar puncture group: Puncture procedures were performed under ultrasound guidance for patients with lesions ≤1 cm and 1 < d ≤ 2 cm in diameter, achieving a 100% success rate (207/207). Preoperative, intraoperative, and postoperative ultrasound images of small liver cancer puncture for patients with different lesion sizes are shown in **Figure 2**. For patients with lesions ≤1 cm in diameter, an average of 3 (3–5) needle passes were performed, yielding 3 (3–5) tissue samples. For patients with lesions 1 < d ≤ 2 cm, an average of 2 (2–4) needle passes were performed, yielding 2 (2–4) tissue samples. The pathological tissue samples from both lesion size groups met the criteria for pathological examination, as shown in **Figure 3**.

Conventional puncture group: Puncture procedures were performed under ultrasound guidance for patients with lesions ≤1 cm and 1 < d ≤ 2 cm in diameter, achieving a 100% success rate (537/537). For patients with lesions ≤1 cm in diameter, an average of 3 (3–5) needle passes were performed, yielding 3 (3–5) tissue samples. For patients with lesions 1 < d ≤ 2 cm, an average of 2 (2–4) needle passes were performed, yielding 2 (2–4) tissue samples.

### Diagnostic performance

Regarding the primary outcome of pathological diagnostic accuracy, the coaxial PDC group demonstrated significantly superior performance compared to the conventional group (**Table 2**). Specifically, for the overall cohort (lesions ≤2 cm) and for lesions measuring 1–2 cm, the diagnostic accuracy was significantly higher in the coaxial PDC group (P = 0.005 and P = 0.002, respectively). For lesions ≤1 cm, although the coaxial PDC group showed a numerically higher accuracy rate, the difference did not reach statistical significance (P = 0.657).

**Table 2:** Comparison of pathological diagnostic accuracy between the two group.

Group	Lesion size	Total, n	Diagnosed, n	Accuracy, %	P Value
Combined coaxial trocar puncture group	Overall (≤2 cm)	207	198	95.6	0.005
Conventional puncture group	Overall (≤2 cm)	537	478	89.0	
Combined coaxial trocar puncture group	1 < d ≤ 2 cm	172	168	97.7	0.002
Conventional puncture group	1 < d ≤ 2 cm	446	403	90.4	
Combined coaxial trocar puncture group	d ≤ 1 cm	35	30	85.7	0.657
Conventional puncture group	d ≤ 1 cm	91	75	82.4	

Diagnostic accuracy = (number of diagnosed cases/total cases) × 100%. Intergroup comparisons were performed using the Chi-square test.

#### Intraoperative and postoperative bleeding

**Figure 4:**
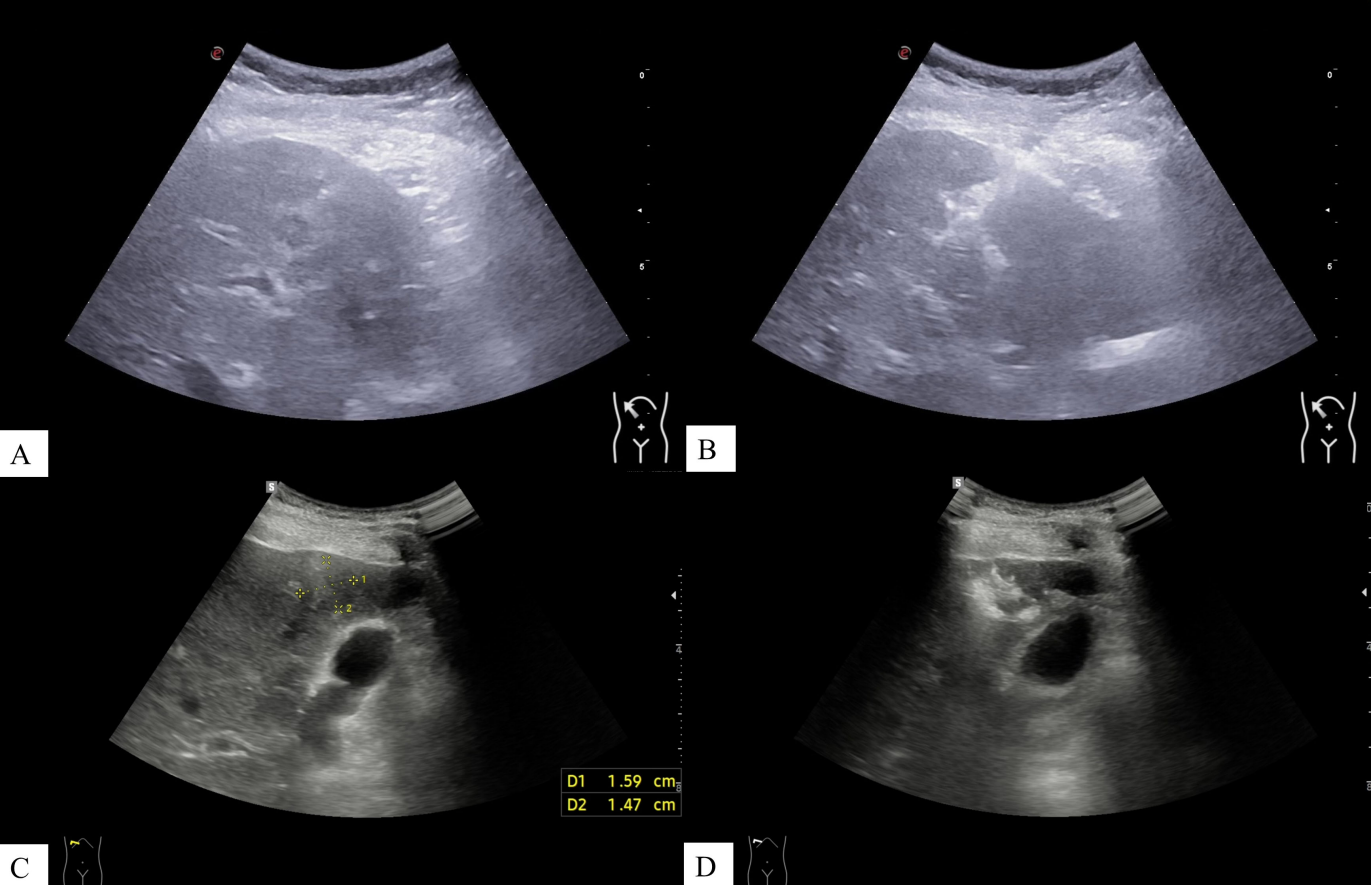
Ultrasound images before and after thrombin injection during combined coaxial trocar puncture in patients with different lesion sizes. **(A, B)** Images from a patient with a lesion diameter ≤1 cm: **(A)** pre-injection, **(B)** post-injection. **(C, D)** Images from a patient with a lesion diameter of 1 < d ≤ 2 cm: **(C)** pre-injection, **(D)** post-injection.

Combined coaxial trocar puncture group: During the puncture, intraoperative bleeding was observed via the coaxial trocar. The incidence of intraoperative bleeding in the group with lesions ≤1 cm was 14.2% (5/35), while in the group with lesions measuring 1 < d ≤ 2 cm, it was 38.9% (67/172). The overall incidence of intraoperative bleeding was 34.7% (72/207). Following the injection of 1–2 KU of thrombin via the coaxial trocar, hemostasis was successfully achieved in all 72 cases, with no instances of postoperative bleeding observed. **Figure 4** illustrates the ultrasound images of a thrombin injection utilized to seal the puncture tract.

Conventional puncture group: No clear intraoperative bleeding was observed during dynamic ultrasound monitoring. However, 5 patients experienced significant bleeding within 2 hours postoperatively, which was successfully managed with interventional embolization.

### Postoperative pain and needle track seeding

Combined coaxial trocar puncture group: 85.0% (176/207) of patients reported no pain (NRS = 0), 15.0% (31/207) reported mild pain (NRS 1-3).

Conventional puncture group: 70.0% (376/537)of patients reported no pain (NRS = 0), 30.0% (161/537) reported mild pain (NRS 1-3).

The overall incidence of any postoperative pain (NRS ≥ 1) was significantly lower in the coaxial group compared to the conventional group. All reported pain was transient and decreased significantly within 1 hour without specific intervention.

No cases of needle tract implantation were observed during the 1-year follow-up after the procedure.

## Discussion

Liver cancer ranks fifth in incidence and second in mortality worldwide, making early diagnosis and treatment essential for improving prognosis (11). Due to the low sensitivity of imaging for lesions ≤2 cm, biopsy is often necessary to confirm the diagnosis and provide a histological basis for clinical decision-making (12).

Transabdominal ultrasound-guided biopsy is a widely used and reliable technique offering real-time vascular visualization, multidimensional imaging, and avoidance of vital structures, ensuring high safety (13). Under the supervision of ultrasound, the biopsy needle can be precisely directed to the target area. According to the 2024 Guidelines for Primary Hepatocellular Carcinoma, small tumor size and deep location reduce biopsy detection rates. For lesions ≤1 cm, the procedure is more challenging and yields smaller tissue samples, which directly affects diagnostic accuracy. Insufficient tissue may lead to misdiagnosis, inaccurate grading, and staging (14). In addition, tumor heterogeneity and sampling errors may lead to false-negative results.

This study demonstrated the efficacy of coaxial needle biopsy, with successful puncture achieved in all patients. The coaxial trocar allows for multiple, multi-angle, and multi-point sampling, facilitating the acquisition of sufficient tissue for accurate pathological diagnosis (14). Previous studies reported diagnostic accuracy of 82-94% for non-coaxial biopsy techniques in small hepatic carcinoma (15). In the present retrospective study analyzed 537 patients with small liver cancer (≤2 cm) who underwent ultrasound-guided non-coaxial needle biopsy at Jiangsu Provincial Cancer Hospital from January 2021 to March 2024, achieving a pathological diagnostic rate of 89%. In comparison, 207 patients biopsied using combined coaxial trocar achieved a higher diagnostic rate of 95.6% (198/207), demonstrating a significant advantage of this technique in detecting small liver cancers (P = 0.005). The established biopsy channel allows the operator to increase sampling frequency by adjusting specimen length, improving diagnostic accuracy, especially for lesions ≤1 cm. Nevertheless, 9 cases remained undiagnosed, possibly due to lesion size (five ≤1 cm) or underlying liver disease. Smaller lesions and chronic liver abnormalities with heterogeneous echogenicity may increase the false-negative rates and complicate tissue sampling.

The complication rate of ultrasound-guided liver lesion biopsy was 5.9%, primarily including pain, bleeding, peripheral organ injury, and needle tract implantation, with pain and bleeding being the most common (16).

A meta-analysis reported mild bleeding complications in 3–4% of ultrasound-guided liver biopsies, with severe bleeding requiring transfusion in about 0.5% of cases (17). In this study, intraoperative bleeding occurred in 72 patients in the coaxial trocar group, all successfully controlled by thrombin injection through the trocar, with no postoperative bleeding observed. This efficacy is attributed to the established biopsy channel and timely thrombin administration. These findings suggest that coaxial trocar biopsy effectively manages intraoperative bleeding and reduces postoperative hemorrhage (18). In contrast, no significant intraoperative bleeding was observed in the conventional group, but 5 patients experienced major postoperative bleeding within 2 hours. This may due to the absence of a biopsy channel, limiting real-time bleeding monitoring and delaying intervention. Moreover, conventional biopsy’s lack of a biopsy tract and precise puncture distance control often requires multiple needle insertions, increasing tissue damage and bleeding risk. The combined approach, with an established tract and accurate distance control, allows multiple insertions without raising bleeding risk.

Once the biopsy tract is established with the coaxial trocar, the operator avoids repeated needle insertions, thereby reducing stimulation of the hepatic peripheral nerves are engaged and minimizing patient pain. By using the coaxial trocar to precisely control the cutting length of the tissue specimen, which helps minimize damage to surrounding normal tissues, further reducing both the incidence and severity of pain.

The combined coaxial trocar biopsy technique allows precise control of the cutting instrument’s distance from the target tissue, ensuring accurate needle placement. This reduces damage to surrounding tissues, minimizes bleeding, and lowers the risk of needle tract implantation. A meta-analysis of 1,340 cases reported a 2.7% incidence of needle tract implantation after liver biopsy (19). Notably, no patients in this study developed needle tract implantation, confirming the safety of combined coaxial trocar biopsy for small hepatic carcinoma. By using a single puncture site through the hepatic peritoneum, this technique protects surrounding tissues from tumor seeding during multiple sampling passes, effectively minimizing implantation risk (20–22).

Beyond procedural safety, patient tolerance is a practical consideration. This study found that the incidence of postoperative pain was significantly lower in patients undergoing the coaxial PDC technique compared to the conventional non-coaxial group. This finding aligns with the technical objective of achieving a minimally invasive procedure through precise distance control. Although all reported pain was mild and transient, the reduction in the frequency of pain episodes contributes to an improved subjective patient experience, which represents a finding of positive clinical significance in its own right (23).

Some studies have suggested that coaxial trocars may be associated with longer retention times and a higher risk of target tissue tearing (24), potentially compromising biopsy performance (25). However, other research shows that combined coaxial trocar biopsy reduces needle insertions, shortens procedure time (26), and that intraoperative thrombin injection into the needle tract effectively lowers postoperative bleeding, making it superior to non-coaxial techniques. In this study, experienced operators instructed patients to maintain steady respiration to minimize liver movement and reduce the risk of tissue tearing.

## Study limitations

This study has several limitations. First, it is a single-center review initiated by the Department of Ultrasound at Jiangsu Provincial Cancer Hospital, relying on retrospective analysis of electronic medical records. Consequently, incomplete records may occur if patients sought care outside our hospital, potentially leading to inaccuracies in the documentation of complications. To further validate our findings and achieve more robust results, large-scale, multicenter studies are warranted. Second, the assessment of postoperative pain, though standardized using the NRS, is inherently subjective. Factors such as individual pain tolerance and anxiety were not quantified and may influence the reported scores. To further validate our findings and obtain more robust evidence, future large-scale, multicenter studies that prospectively control for these confounding factors are warranted.

## Conclusion

In this study, we evaluated a PDC strategy for ultrasound-guided coaxial biopsy of small hepatic carcinomas (≤2 cm). Compared to conventional non-coaxial biopsy, the PDC-enhanced coaxial technique achieved a significantly higher diagnostic yield while maintaining a favorable safety profile, characterized by a lower incidence of postoperative pain and no observed needle-tract implantation.

Therefore, the PDC strategy represents a safe, effective, and clinically valuable refinement of coaxial biopsy, providing a systematic approach to enhance precision. It has the potential to improve diagnostic confidence and patient outcomes in the management of challenging small liver tumors.

## Data Availability

The original contributions presented in the study are included in the article/supplementary material, further inquiries can be directed to the corresponding author/s.
