# Correction: Precision distance control in ultrasound-guided coaxial needle biopsy for small liver cancer: improving safety and diagnostic accuracy yield

**DOI:** 10.3389/fonc.2026.1835624

**Published:** 2026-04-24

**Authors:** Yaqi Zhang, Qian Huang, Lilin Zhao, Ting Zhang

**Affiliations:** Department of Ultrasound, The Affiliated Cancer Hospital of Nanjing Medical University, Jiangsu Cancer Hospital, Jiangsu Institute of Cancer Research, Nanjing, Jiangsu, China

**Keywords:** coaxial needle, precision distance control, procedure - related complications, small component, small liver cancer

Authors Yaqi Zhang, Qian Huang and Lilin Zhao were erroneously assigned as equal contributing authors.

