# Correction: Precision distance control in ultrasound-guided coaxial needle biopsy for small liver cancer: improving safety and diagnostic accuracy yield

**DOI:** 10.3389/fonc.2026.1871904

**Published:** 2026-05-12

**Authors:** Yaqi Zhang, Qian Huang, Lilin Zhao, Ting Zhang

**Affiliations:** Department of Ultrasound, The Affiliated Cancer Hospital of Nanjing Medical University, Jiangsu Cancer Hospital, Jiangsu Institute of Cancer Research, Nanjing, Jiangsu, China

**Keywords:** coaxial needle biopsy, diagnostic accuracy, precision distance control, small liver cancer, ultrasound-guided

The funder “Jiangsu Provincial Key Research and Development Program, Grant No. BE2021711” to Ting Zhang was erroneously omitted.

The original version of this article has been updated.

